# Evaluation of the effect of intrathecal clonidine to decrease shoulder tip pain in laparoscopy under spinal anaesthesia

**DOI:** 10.4103/0019-5049.65370

**Published:** 2010

**Authors:** Poonam S Ghodki, Shalini P Sardesai, Shalini K Thombre

**Affiliations:** Lecturer, Department of Anaesthesiology, Shrimati Kashibai Navale Medical College and General Hospital, Narhe, Pune, India; 1 Associate Professor, Department of Anaesthesiology, Shrimati Kashibai Navale Medical College and General Hospital, Narhe, Pune, India; 2 Professor and HOD, Department of Anaesthesiology, Shrimati Kashibai Navale Medical College and General Hospital, Narhe, Pune, India

**Keywords:** Bupivacaine, clonidine, intrathecal, laparoscopy, shoulder tip pain

## Abstract

Sixty ASA grade I/II patients scheduled for elective short laparoscopic procedures under spinal anaesthesia were divided into two groups of 30 each. The first group (group C) received 3.5 ml of hyperbaric bupivacaine with 30 mcg of clonidine. The second group (group B) received plain bupivacaine 3.5 ml. Till date, the limiting factor for use of spinal anaesthesia for laparoscopy was patient’s discomfort due to shoulder tip pain. From our study it can be concluded that bupivacaine along with clonidine in low doses provides good sedation and analgesia in intraoperative and post-operative period and at the same time abolishes shoulder tip pain during laparoscopic procedures. In addition, no significant changes in haemodynamics occur with the low dose of clonidine used.

## INTRODUCTION

Laparoscopic surgeries are very commonly performed surgeries and they offer several advantages over open laparotomies. Although general anaesthesia is considered the choice of anaesthesia for laparoscopy, regional anaesthesia in the form of spinal anaesthesia provides unique advantages over general anaesthesia.[1–3] The only limiting factor for use of spinal anaesthesia in laparoscopy is patient’s discomfort with pneumoperitoneum and the associated shoulder tip pain.[4–6]

Several studies have been conducted to find ways for decreasing shoulder tip pain in laparoscopy under spinal anaesthesia.[7–10] Intrathecal opioids have been tried in combination with local anaesthetics for spinal anaesthesia but post-operative nausea, vomiting, and pruritis have been the limiting factor for their use. Studies have been conducted using intrathecal clonidine in doses up to 1 mcg/kg.[11–13] These doses are associated with sympatholytic side effects of clonidine along with analgesic effects disfavouring its use. No study has been conducted to evaluate the effect of intrathecal clonidine in the concentration of 30 mcg for laparoscopy and its efficacy to attenuate shoulder tip pain. In our study we found that local anaesthetics in combination with low-dose clonidine not only have synergistic effect but also decrease shoulder tip pain and provide good sedation alleviating the need of intravenous analgesics and sedation.

We decided to compare the effect of spinal anaesthesia with bupivacaine alone to bupivacaine with low-dose clonidine on haemodynamics, duration of spinal anaesthesia and quality of post-operative analgesia, intra- and post-operative sedation, incidence of PONV and complications if any and at the same time to assess the efficacy of intrathecal clonidine as a remedy to shoulder tip pain in laparoscopy.

## METHODS

The present study was conducted after obtaining written, informed and valid consent. Approval from the institutional ethics committee was obtained for the study.

Inclusion criteria were as follows:

1. ASA grade I and II patients.
2. Age: 18–50 years, either sex.
3. Surgery: Elective short laparoscopic procedures with estimated pneumoperitoneum time less than 60 min, e.g. laparoscopic appendicectomy, cholecystectomy, ovarian cystestomy, lymph node biopsy, diagnostic laparoscopy.

Exclusion criteria were as follows:

1. ASA grade > II
2. Age: < 18 or >50 years
3. Surgery: Prolonged laparoscopic procedures like laparoscopy-assisted vaginal hysterectomy, laparoscopic fundoplication and laparoscopic nephrectomy.
4. Patients’ refusal.
5. Known contra-indications to spinal anaesthesia.

All patients were examined a day before surgery and were kept fasting overnight. They received inj. Glycopyrrolate 0.2 mg and inj. Ondansetron 4 mg intravenously as premedication. In the operation theatre, patient’s baseline pulse, blood pressure, saturation, etCO_2_, respiratory rate, and ECG were recorded and all were preloaded with ringer lactate 15 ml/kg. These patients were randomly assigned using a sealed envelope technique into two groups in a double-blind manner. Group B (*n* = 30) received 3.5 ml of heavy bupivacaine and group C received 3.3 ml of bupivacaine along with 30 mcg to make a total volume of 3.5 ml.

Spinal anaesthesia was given in a sitting position in L3–L4 interspace with 26 gauge Quincke’s needle. The anaesthesiologist giving spinal anaesthesia was blinded to the solution administered intrathecally. Patients were made supine and following parameters noted: time of the onset of sensory block as assessed by pinprick, onset of motor block assessed by using Bromage scale, time to achieve maximum sensory level, two-segment regression and the time when patient demanded rescue analgesia. The operating table was adjusted to achieve a sensory level of maximum T4 in all patients. Intraoperative pulse, BP, RR, sPO_2_, etCO_2_ by a side stream capnometer placed at the nostril and ECG were monitored and noted at the time of induction, post-induction, during the creation of pneumoperitoneum, then every 15 min throughout the procedure and every 30 min post-operatively. Inflation pressure of CO_2_ during pneumoperitoneum was kept below 15 mmHg in all cases. The quality of sedation was determined using the Ramsay scale. Hypotension was defined as >20% decrease in systolic blood pressure and was treated with intravenous fluids and ephedrine 6 mg in incremental doses. Bradycardia (HR< 60/min) was treated with intravenous atropine. Intraoperative complaints of shoulder tip pain were noted and the severity was gauged using the 10 cm visual analogue scale (VAS). Adverse effects such as PONV were also recorded. No other sedative or analgesic was given intraoperatively until demanded by the patient. When demanded, sedation was given in the form of inj. Midazolam 0.03 mg/kg iv. and analgesia in the form of inj. Pentazocine 0.3 mg/kg iv. Post-operative rescue was given in the form of diclofenac sodium 75 mg intravenous infusion. The time when patient demanded rescue analgesic was also recorded. The duration of spinal anaesthesia was considered as that from the onset of sensory block to the administration of the rescue analgesic.

Data were analyzed using statistical tests, chi-square and Student’s *t*-test; *P* < 0.05 was considered statistically significant.

## RESULTS

A total of 60 patients undergoing elective laparoscopic surgeries under spinal anaesthesia were enrolled in our study which were divided into two groups, namely group B receiving 0.5% hyperbaric bupivacaine 3.5 ml and group C receiving 0.5% hyperbaric bupivacaine 3.3 ml along with inj. clonidine 30 mcg to make a volume of 3.5 ml. Sample size calculation was done using statistical software (Epi Info software, version 3.2). Both groups were comparable with respect to age, sex and weight [Table 1].

**Table 1:** Demographic data of patients

	Group B		Group C		*P* value
	Mean	SD	Mean	SD
Age(yrs)	32.40	12.37	38.43	15.82	0.1052
Weight (kg)	52.37	9.11	50.40	9.14	0.4073
M:F		6:24		9:21	0.3711

SD- standard deviation, M:F- Male: Female.

**Figure 1:**
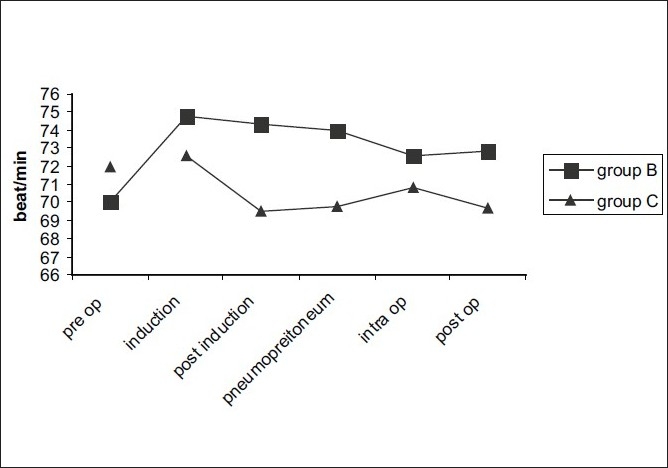
Comparison of pulse rate between the two groups

Figure 1 shows comparison of pulse rates between the two groups. The pulse rate was maintained in group B; however in group C, a slight fall in the pulse rate from baseline was recorded with the lowest pulse rate being 60/min. The difference in pulse rates between the two groups was just statistically significant with a *P*-value of 0.043. However, none of the patients in either group needed atropine.

Blood pressure in both the groups increased after the creation of pneumoperitoneum with the mean value of 140/88 mmHg in group B and 136/80 mmHg in group C. However, later in the intraoperative period, there was a fall in BP in both the groups. In group C, two patients and in group B, one patient required ephedrine treatment. Rest of the BP changes in the intra- and post-operative period were comparable with a *P*-value of 0.37 making it statistically insignificant. EtCO_2_ increased in response to pneumoperitoneum in both the groups and changes were comparable (*P*-value – 1.00). Saturation and ECG were normal in both the groups throughout the procedure and post-operatively.

The quality of spinal anaesthesia is compared in Table 2 from where it can be discerned that clonidine has no effect on the onset of spinal anaesthesia. The sensory and motor level onsets were similar in either group. Two segment regression occurred early in group B than group C and the difference was statistically significant (*P* = 0.001). In addition, it was noted that clonidine significantly prolongs the duration of spinal anaesthesia thus extending the analgesia as indicated by delayed demand for rescue analgesia in the post-operative period.

**Table 2:** Onset and duration of spinal anaesthesia and time of rescue analgesia

	Group B	Group C	*P* value	Significance
Onset of SA (min)	9.4	9.46	0.877	NS (t test)
Duration of SA (min)	220	311	0.00	S (t test)
Time of rescue analgesia (min)	195.5	261.5	0.00	S (t test)

SA – spinal anaesthesia, NS – not significant, S – significant

In our study we found that in group C, two patients complained of shoulder tip pain. However, 27 patients in group B had shoulder tip pain and sedation and analgesia for obviating discomfort was demanded by 26 patients [Table 3]. These data were analysed by chi-square analysis corrected by the Yates method and the difference in complaints of shoulder tip pain as well as sedation-analgesia requirements in both groups were found to be statistically significant.

**Table 3:** Incidence of shoulder tip pain and requirement of sedation-analgesia

	Group B	Group C	Significance
Shoulder tip pain	27	2	S
Sedation and analgesia requirement	26	2	S

S - significant

None of the patients of either group had PONV or any other complication.

## DISCUSSION

Laparoscopic surgeries under spinal anaesthesia are generally complicated by shoulder tip pain.[5,6] The exact aetiology of this shoulder tip pain is not known although several studies have been put forward for its explanation, the most popular one being that of diaphragmatic irritation and the shoulder tip pain being actually a referred pain and hence difficult to treat. In our study we evaluated the efficacy of low-dose intrathecal clonidine to abolish the shoulder tip pain.

Clonidine is a selective alpha 2 agonist and acts by inhibiting norepinephrine release from presynaptic terminals. This effect is responsible for the sympatholytic effect produced by clonidine.[14] It produces sedation and analgesia by its action on spinal cord and locus ceruleus.[15] Analgesia that is produced by clonidine is not only because of sympatholysis at peripheral level, but also due to decrease catecholamine release in brain. This results in an overall analgesic effect of clonidine. Also, it has been shown that intrathecal clonidine suppresses tumour necrosis factor a in plasma and CSF during the perioperative period which is presumed to result in analgesia.[16] All these effects may be responsible for decreasing the shoulder tip pain in laparoscopy.

Intrathecal clonidine has been shown to prolong the action of spinal anaesthesia when added to local anaesthetic.[12,13] It has also been seen to provide better post-operative analgesia after intrathecal use.[17] Sympatholytic effects of intrathecal clonidine resulting in hypotension are seen with higher doses forcing the use of vasopressors.[18] In our study, we found that a low dose of intrathecal clonidine of 30 mcg does not result in worrisome hypotension. This can be attributed to the low dose used and also to the increased peripheral vascular resistance after pneumoperitoneum creation which counteracts the fall in blood pressure. However, bradycardia does occur with this dose, but seldom requires treatment. There are no episodes of apnea, pruritus or PONV as may be seen with opioids when they are used as intrathecal adjuvants with a local anaesthetic.[19,20] The sedation provided by clonidine is of good quality as elicited by the Ramsay score. This decreases the requirement of additional intravenous drugs. The combined property of sedation and analgesia of clonidine keeps patients pain free and comfortable. At the same time this obviates unnecessary intravenous interventions, thus serving the purpose of spinal anaesthesia. General anaesthesia in laparoscopy may be preferred in laparoscopy with prolonged pneumoperitoneum times or that involving diaphragm like laparoscopic fundoplication for hiatus hernia. Spinal anaesthesia not only provides good muscle relaxation and optimum operative field, but also facilitates better post-operative analgesia. And with adjuvants like low-dose intrathecal clonidine, stable haemodynamics along with good sedation and analgesia is provided abolishing shoulder tip pain, making the procedure more economical and comfortable to the patient.

We therefore recommend the use of low-dose clonidine along with bupivacaine in spinal anaesthesia for short-duration laparoscopic surgeries.
